# The morphology of the preimaginal stages of *Rhinusaneta* (Germar, 1821) and notes on its biology (Coleoptera, Curculionidae, Mecinini)

**DOI:** 10.3897/zookeys.807.28365

**Published:** 2018-12-17

**Authors:** Radosław Ścibior, Jacek Łętowski

**Affiliations:** 1 Department of Zoology, Animal Ecology and Wildlife Management, University of Life Sciences in Lublin, Akademicka 13, 20-950 Lublin, Poland University of Life Sciences in Lublin Lublin Poland

**Keywords:** Egg, host plant, life development, *
Linaria vulgaris
*, mature larva, parasitoid, Plantaginaceae, pupa, weevil

## Abstract

A detailed description of the mature larva and pupa of *Rhinusaneta* (Germar, 1821) and new diagnostic features of this species are presented. The development cycle of *R.neta* in the standard conditions lasts almost 60 days: an 11-day egg period, a 29-day larval period, and an 18-day pupal period, on average. The larvae are parasitised by hymenopterans of the superfamily Chalcidoidea. Similarities and differences with *Rhinusabipustulata* and other species of this genus are presented.

## Introduction

The taxon *Rhinusa* attained the rank of the genus based on the classification made by Caldara (2001). It belongs to the tribe Mecinini and includes 40 species around the world (Caldara et al. 2014). In Poland, ten species have thus far been recorded (Petryszak 2004; Mokrzycki and Wanat 2005), of which two, *R.hispida* (Brullé, 1832) and *R.thapsicola* (Germar, 1821), were recognised by Caldara (2013) as synonymous with *R.tetra* (Fabricius, 1792). The known host plants of *Rhinusa* species include plants only from the families Plantaginaceae and Scrophulariaceae (Caldara et al. 2010; Caldara et al. 2014; APG 2016). Weevils inhabit mainly warm habitats, such as pastures, sunlit slopes, sandbars, gravel pits, uncultivated strips between fields, roadsides, etc. (Koch 1992; Burakowski et al. 1997). Till now the data on the morphology and biology of preimaginal stages (in varying degrees of detail) are available for the following species: *R.asellus* (Gravenhorst, 1807), *R.antirrhini* (Paykull, 1800), *R.bipustulata* (Rossi, 1792), *R.collina* (Gyllenhal, 1813), *R.linariae* (Panzer, 1795), *R.neta*, *R.pilosa* (Gyllenhal, 1838) and *R.tetra* (Emden 1938; Scherf 1964; Anderson 1973; Burakowski et al. 1997; Gosik 2010a; Gassmann et al. 2014).

*Rhinusaneta* is a species distributed mainly in southern and central Europe, also noted in Belgium, the Caucasus, Afghanistan, Kazakhstan, Iran, Algeria, and Morocco (Burakowski et al. 1997, Caldara 2013). In Poland, it is found in twelve geographical realms in the lowlands and highlands (mainly in central and eastern Poland), as well as in some lower locations in the Eastern Sudetes and the Eastern Beskids. It inhabits dry, warm places in fields and on fallow land, roadsides, gravel pits, moors and rock faces, as well as in sunny thickets. It develops on *Linariavulgaris* (L.) Mill., *L.genistifolia* (L.) Mill., *L.repens* (L.) Mill., *L.spuria* (L.) Mill., *Antirrhinumorontium* L. and *A.majus* L. (Plantaginaceae). Adults are active on plants from May to August. The larva feeds inside seed capsules on immature seeds (Smreczyński 1976, Burakowski et al. 1997). Feeding by the larvae does not cause swelling in the seeds, as in the case of feeding by the larvae of *R.antirrhini*. In North America, in laboratory conditions, it has also fed on species of the genera *Chaenorrhinum* and *Kickxia* (Plantaginaceae), but in field conditions only on *L.vulgaris* and *L.dalmatica* (Wilson et al. 2005).

*Rhinusaneta* was introduced to the United States in 1937 and to Canada in 1957, and from the late 1950s, it was used in both countries for biological control of two species of toadflax (*L.vulgaris* and *L.dalmatica*). The primary host plant for *R.neta* in North America is *L.dalmatica* and the secondary host plant is *L.vulgaris*. *R.neta* is much less common in these countries and is found in more dispersed populations than the morphologically very similar *R.antirrhini*. When both species feed on *Linaria* at the same time, seed losses may reach 90% (Wilson et al. 2005).

The aim of present paper is to describe development stages (mature larva L_3_ and pupa) of *Rhinusaneta* and collect a new diagnostic features of this species. Some data about biology are also presented.

## Materials and methods

### Collection in the field

Samples with flowering and fruiting *Linariavulgaris* plants were collected from June to August in 2014–2016 in the following locations in the Lublin Upland (south-eastern Poland): Garbów (51°22'15.91"N, 22°21'25.82"E), unplowed strip between fields; Lublin (51°13'39.15"N, 22°38'31.43"E), lawn next to the street; Lublin (51°14'42.21"N, 22°32'12.93"E), ruderal vegetation next to tennis courts (UMCS campus); Niebrzegów (Bonów) (51°30'48.28"N, 21°57'03.43"E), meadow; and Mięćmierz (51°18'22.50"N, 21°54'15.80"E), unplowed strip between fields. Adults were collected from plants using a sweep net in series of 100 sweeps each time, at intervals of 3–7 days, from May to August on sunny, windless days, during the hours (10 am to 4 pm) where they are most active. To obtain preimaginal stages, the aboveground parts of *Linariavulgaris* were collected for further detailed analysis in the laboratory. In total, ca. 520 specimens of the host plant were collected in the field and examined. The development stages (eggs, larvae, and pupae) were isolated from plants by making delicate cuts in the developing infructescence and extracting them from its interior. Some of the eggs and larvae (ten of both stages) were used for further breeding in the insectarium, and others were used for measurements (also ten) and microscope slides.

### Breeding

Developmental stages (L_3_) isolated from the generative parts were transferred to Petri dishes on an ongoing basis. Further breeding was carried out in accordance with recommendations by Scherf (1964) and Łętowski (1991). The Petri dishes were placed in an incubation chamber under the following conditions: temperature 25 °C during the day and 20 °C at night, humidity 40% during the day (16 h) and 50% at night (8 h). The humidity inside the Petri dishes was higher, 65–80%.

### Graphics

Preparation of microscope slides of preimaginal stages and graphics. Larvae and pupae were treated with lactic acid C_3_H_6_O_3_ (80%). Smaller morphological structures (mouthparts) were first immersed in a cold KOH solution (5%) before being transferred to lactic acid. Developmental stages were analysed and documented using an OLYMPUS BX61 microscope at magnifications from 200× to 400×. All graphics for the study were prepared using CorelDraw X8 software. Metric data are means from ten measurements. Drawings of the morphological structures of the larva and pupa were based on microscope slides prepared according to Łętowski (1991), Gosik (2010a), and Gosik et al. (2010). The nomenclature of larval (L_3_) and pupal chaetotaxy was based mainly on the work of Marvaldi (2003), Oberprieler et al. (2014) and in the case unnamed structures the also on the work of Skuhrovec and Volovnik (2015). The morphology of the egg, L_3_ and pupa and the developmental cycle from egg to adult were described as well. The biological material is stored in ethyl alcohol C_2_H_5_OH (70%) at the Department of Zoology, Animal Ecology and Wildlife Management, University of Life Sciences in Lublin.

Setae of the thorax and abdomen of the larva (L_3_) and pupa are described for one side only.

## Results

### Rhinusa neta

Taxon classificationAnimaliaColeopteraCurculionidae

(Germar, 1821)

#### Description of egg.

*Measurements* (in mm). Egg length 0.47–0.51 (mean 0.49), width 0.25–0.29 (mean 0.27).

*General.* Oval, slightly oblate.

*Colouration.* Light yellow, smooth surface.

#### Description of mature larva.

**Figure 1.**
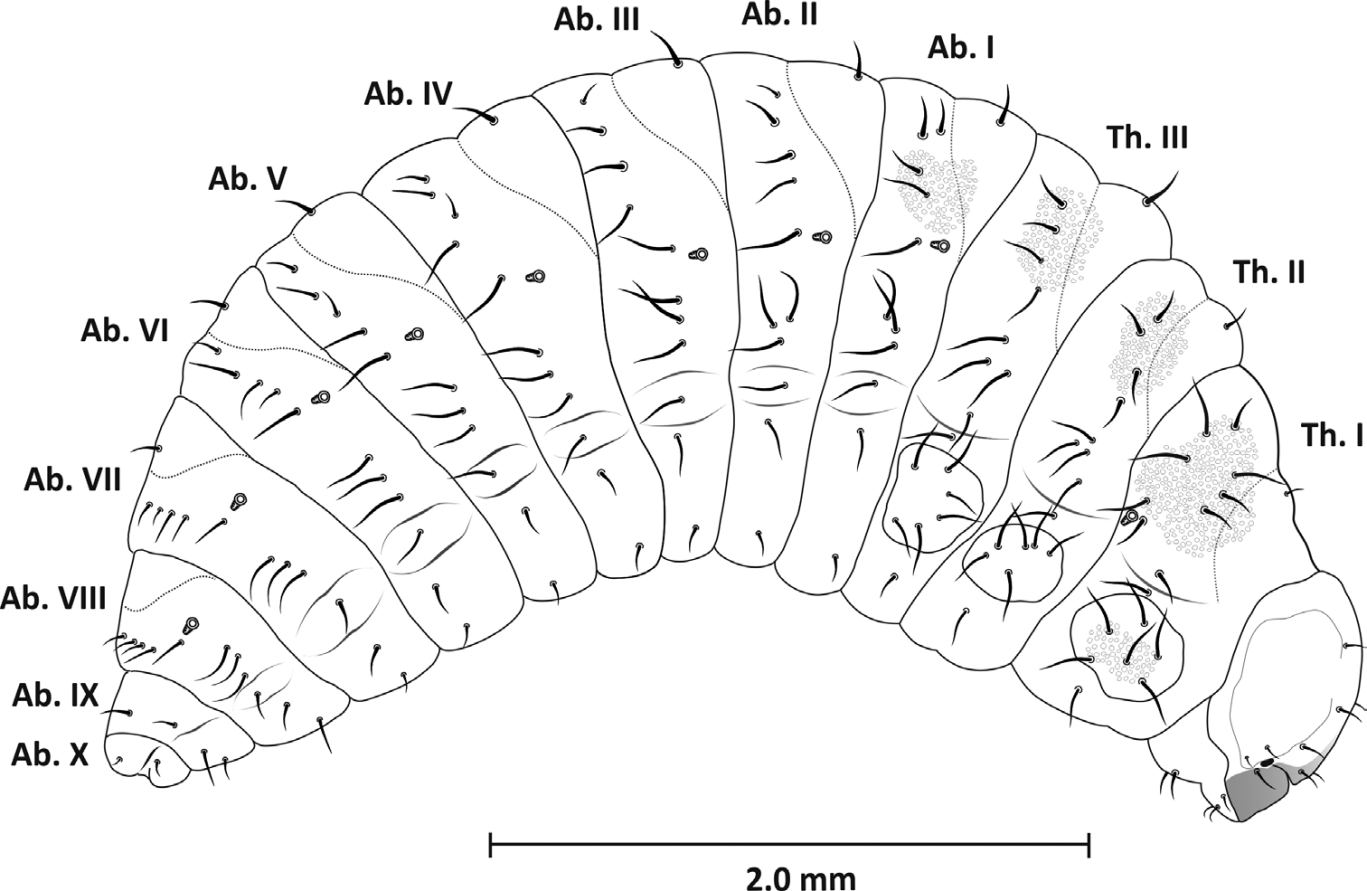
Mature larva (L_3_), lateral view.

(Figure 1, Table 1) *Measurements* (in mm). Body length: 4.40–4.50 mm (mean 4.46). The widest part of the body (Ab. I) 1.73–1.8 (mean 1.76). Epicranium: length 0.54–0.59 (mean 0.57), width 0.58–0.63 (mean 0.61).

*General.* Body massive, strongly curved.

*Colouration.* Head yellow-brown, body light yellow, covered with black, numerous setae.

**Table 1.** Differences of the mature larva (L_3_) of *Rhinusaneta* and *R.bipustulata* based on the publications of Scherf 1964, Anderson 1973, Gosik 2010a, and on personal research.

	* Rhinusa neta *	* Rhinusa bipustulata *
Frontal suture of epicranium	V-shaped	Y-shaped
Endocarina	reaches 4/5 of frons	reaches 1/2 of frons
Setae of head	*des* – 5, *pes* – 4	*des* – 4, *pes* – 5
Stammata (ocelli)	well visible, larger	poorly visible, smaller
Antennae	basal membranous area with 2 sensillae	basal membranous area with 7 sensillae
Labrum, clypeus	clypeus with *2 cls* and 1 sensilla	clypeus with 3 *cls* and no sensilla
Mandible	*mds* – 3	*mds* – 2
Maxillae	basal segment with 1 setae and 2 sensilla, distal segment with group of 10 short cuticular apical processes and 1 sensilla,	basal segment with 1 seta and 1 sensillum, distal segment with group of 4 long cuticular apical processes and 1 sensilla,
Labial complex	prelabium oval, with 2 processes in basal part, *ligs* 2 pair in 2 rows, labial palps one-segmented with 5 conical cuticular apical processes (4 short, 1 long)	prelabium heart-shaped, with 1 process in basal part, *ligs* 2 pair in 1 row, labial palps one-segmented with 5 conical cuticular apical processes (5 short)
Thorax (one side)	prothorax: *prns* on strongly sclerotised shield	prothorax: *prns* not on strongly sclerotised shield
Abdomen (one side)	8 unicameral spiracles located in middle of segments I–VIII, segments I–VII with 1 *prs*, 3 *dpls*, segment VIII with 1 *dls*, 3 *dpls*	8 unicameral spiracles located at anterior margin of segments I–VIII, segments I–VII with 2 *prs*, 1 *dpls*, segment VIII – 1 *prs*, 1 *dpls*

*Vestiture.* Cuticle with strongly chitinised spots in several places on dorsal side. Asperities of body integument present on the surface of all thoracic segments and first abdominal segment covering area occupied by a group of setae *prns* and *pds.* Analogous structure presents only on the surface of the first pedal lobe.

**Figure 2.**
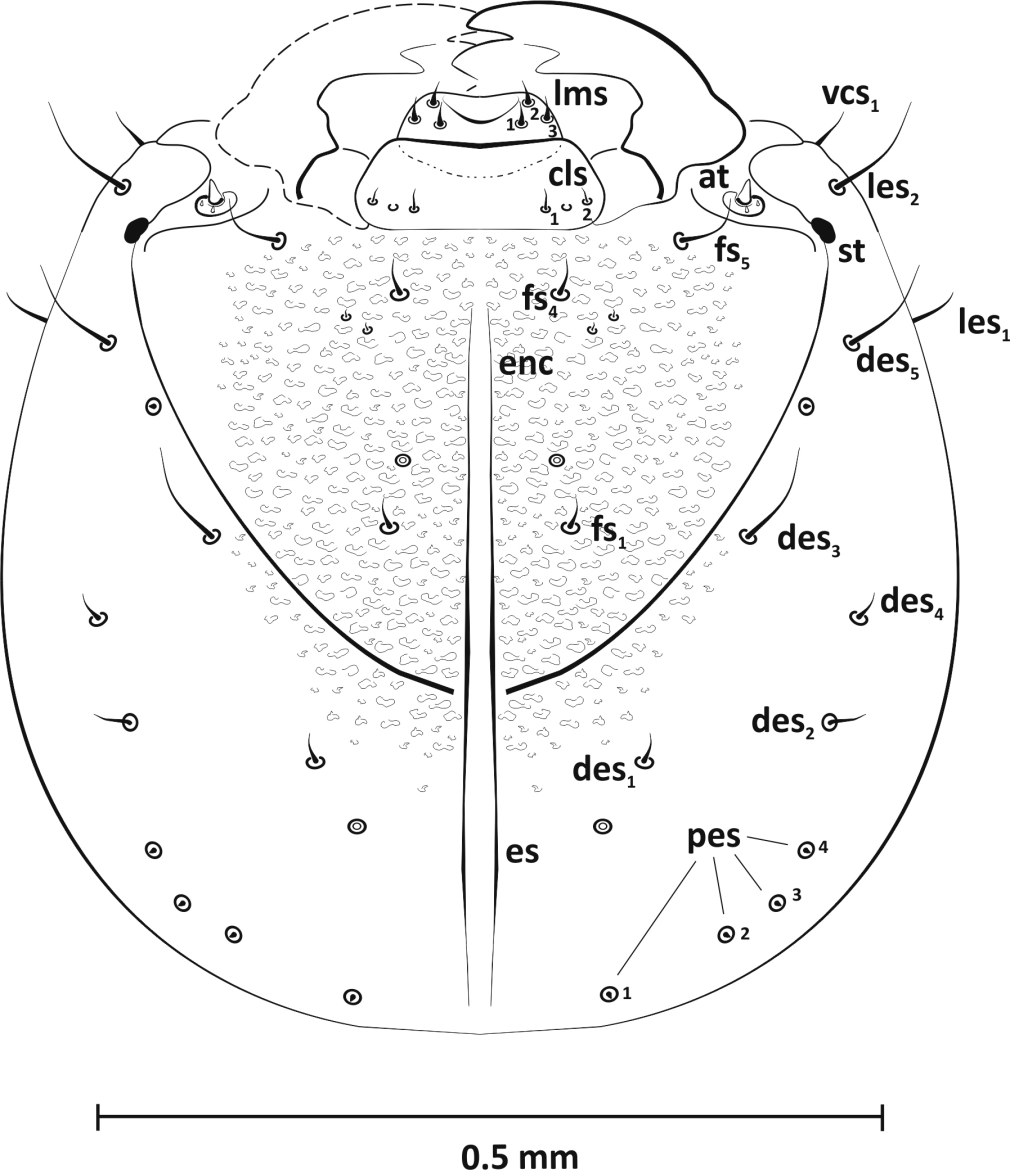
Mature larva (L_3_), epicranium, dorsal view: *at* – antenna, *cls* – clypeal seta, *des* – dorsal epicranial seta, *enc* – endocarina, *es* – epicranial suture, *fs* – frontal seta., *les* – lateral epicranial seta, *lms* – labral seta, *st* – stemmata, *pes* – posterior epicranial seta, *vcs* – ventral cranial seta.

*Head capsule* (Figure 2): Head slightly oval. Endocarina (*enc*) long, nearly 4/5 of frons length. One stemma (*st*) located at end of frontal suture at height of *fs5*. Frons with three distinct setae: longest (*fs5*) at base of antennae, *fs1,4* located in the upper and lower part of frons near endocarina, and two small sensillae (unnumbered) close to *fs4*, which may be highly reduced setae *fs2* and *fs3*. Dorsum of epicranium with five setae (*des1–5*); *des3,5* longest and *des1,2,4* of intermediate length. Posterior epicranium with four small setae (*pes1–4*) arranged in arc not far behind *des2*. On anterior epicranium two long setae *les1–2* protrude from sides and visible *vcs.* Antennae (*at*) with conical sensorium and two very small spinose sensillae.

*Clypeus* with two setae of equal length (*cls1, 2*) at base with one sensilla between them.

**Figure 3.**
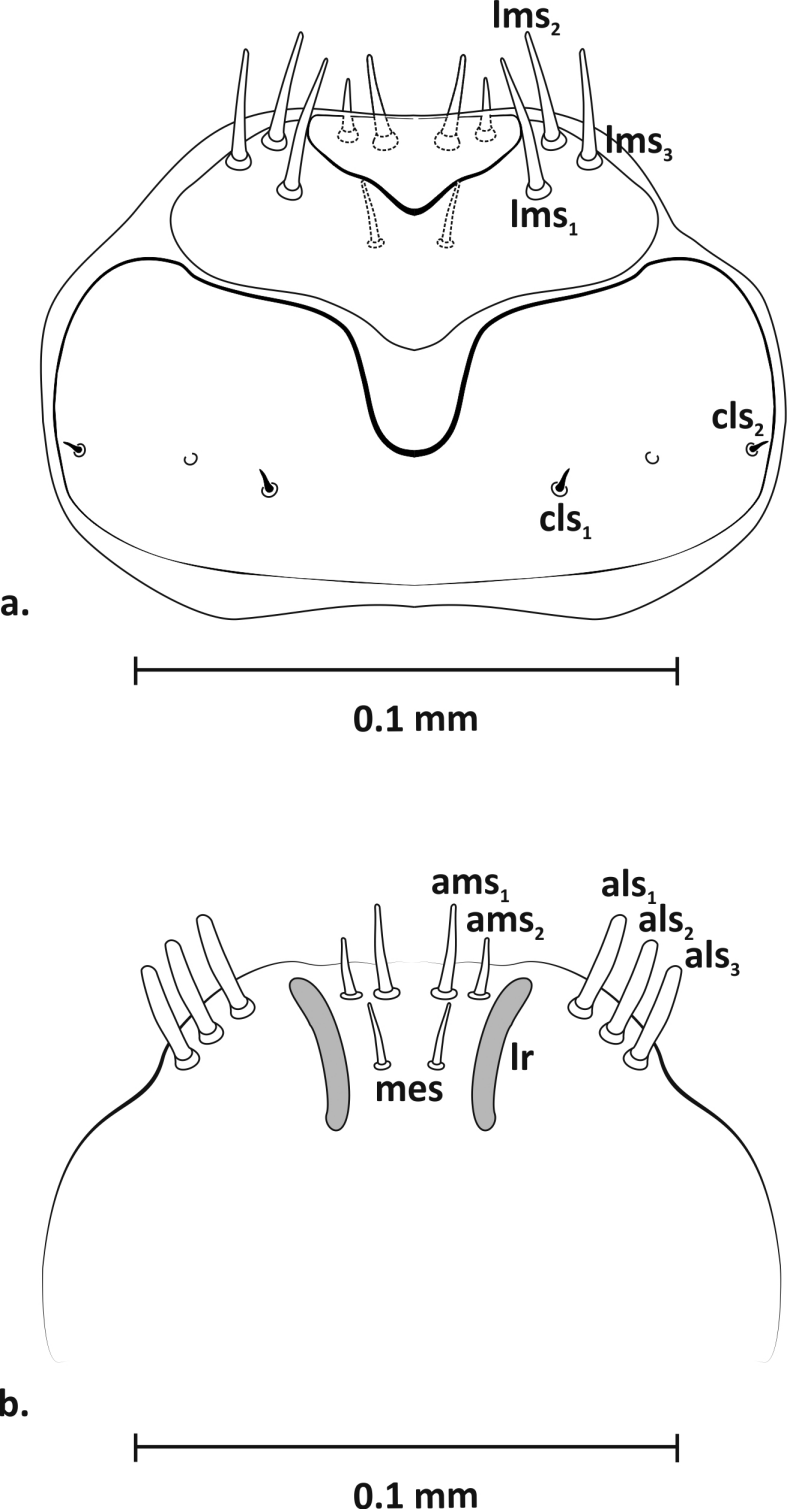
Mature larva (L_3_), clypeus and labrum, dorsal view (**a**): *cls* – clypeal seta, *lms* – labral seta; epipharynx, ventral view (**b**): *als* – anterolateral epipharyngeal seta, *ams* – anteromedian epipharyngeal seta, *lr* – labral rod, *mes* – median epipharyngeal seta.

**Figure 4.**
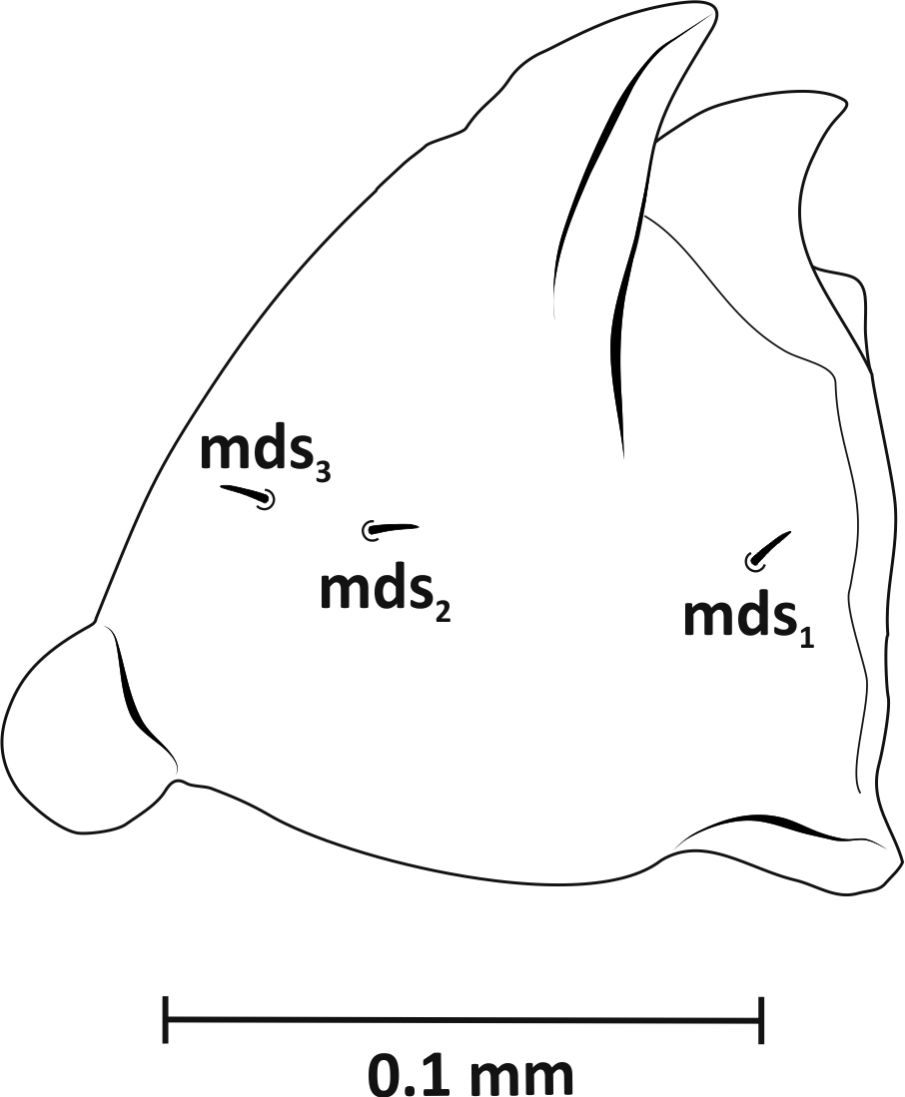
Mature larva (L_3_), left mandible, dorsal view: *mds* – mandibular dorsal seta.

**Figure 5.**
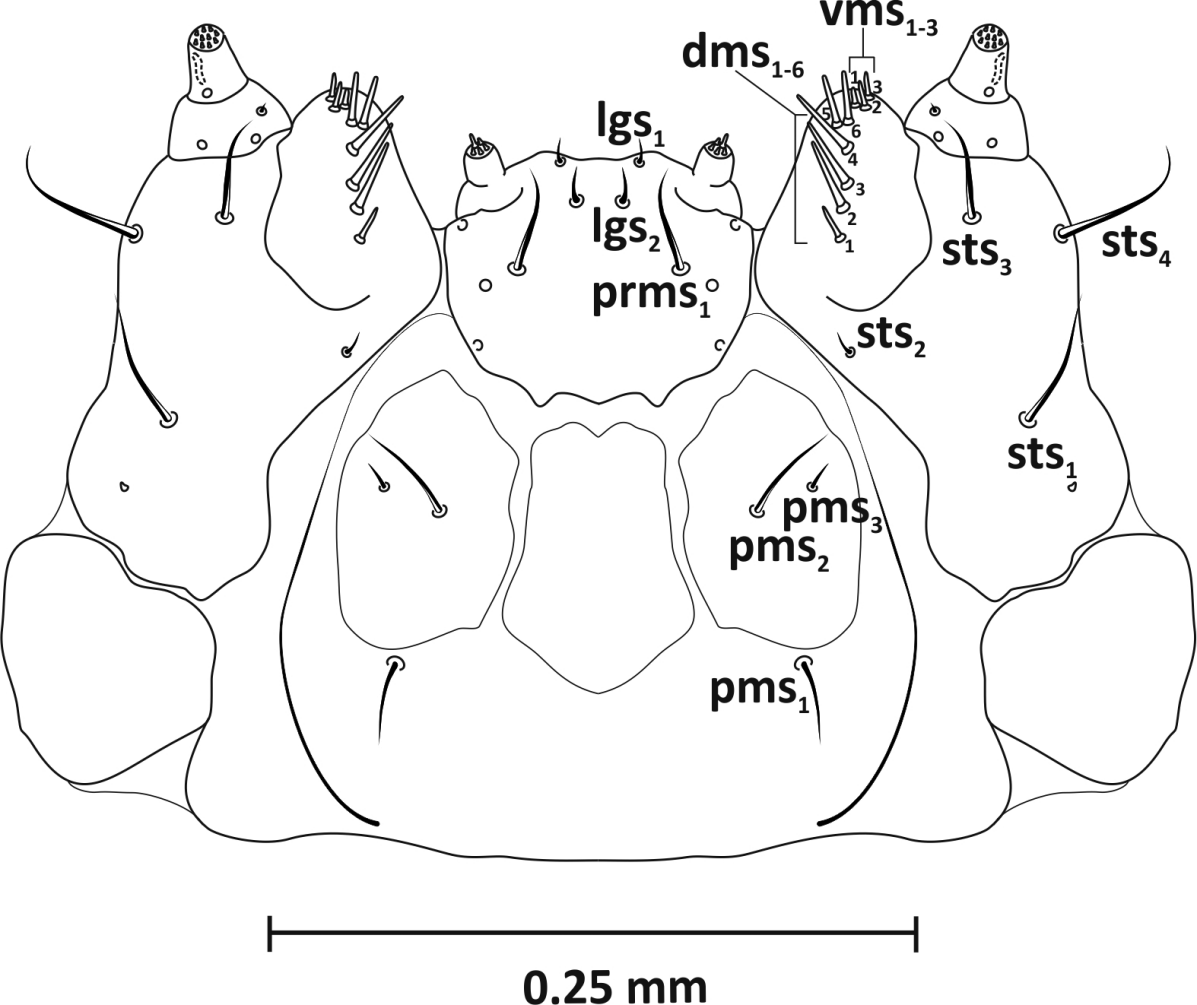
Mature larva (L_3_), maxillae and labium (L_3_): *dms* – dorsal malar seta, *lgs* – ligular seta, *sts* – stipital seta, *pms* – postmental seta, *prms* – premental seta, *vms* – ventral malar seta.

*Mouthparts* (Figs 3–5): Dorsal side of labrum (Figure 3a) ca. 0.09 mm wide with three pairs setae (*lms1–3*). Longest *lms1*, setae *lms2, 3* of similar length, only slightly shorter than *lrms1*. Anterior margin of epipharynx arched, with three thick setae laterally (*als1–3*), two setae (*ams1–2*) on anterior margin, and one seta (*mes*) medially, between well-formed, arcuate labral rods (*lr*) (Figure 3b). Mandibles (Figure 4) massive, red-brown, with two large teeth and one small tooth on inner side; medially on mandible three short setae (*mds1–3*) in one line. Maxillae (Figure 5) yellowish. Palpifer with one long, medially located seta *sts1*, two apically placed setae of equal length *sts3–4* (or *pfs* 1–2) and one small *sts2* (or *mbs*) below molar part. Maxillary palpus with two segments; basal segment with one very short seta (*mxps*) and two sensillae, distal segment cylindrical with accessory process on dorsal side and ten conical papillae. Mala with six setae of unequal length (*dms1–6*), arranged in one line, comb-like, and separate group of three setae (*vms1–3*) situated apically. Labium: postmentum with three setae of unequal length (*pms1–3*) – longest *pms2* and shortest *pms3*. Prementum with two distinct teeth on basal part, three setae (*prms1* and *lgs1–2*) and three sensillae. Seta *prms1* very long, more than three times longer than *lgs2*. One pair *lgs1* on apex and with them slightly larger pair *lgs2* placed closer together. Labial palpus 1-segmented, with four shorter and one longer papilla located apically.

**Figure 6.**
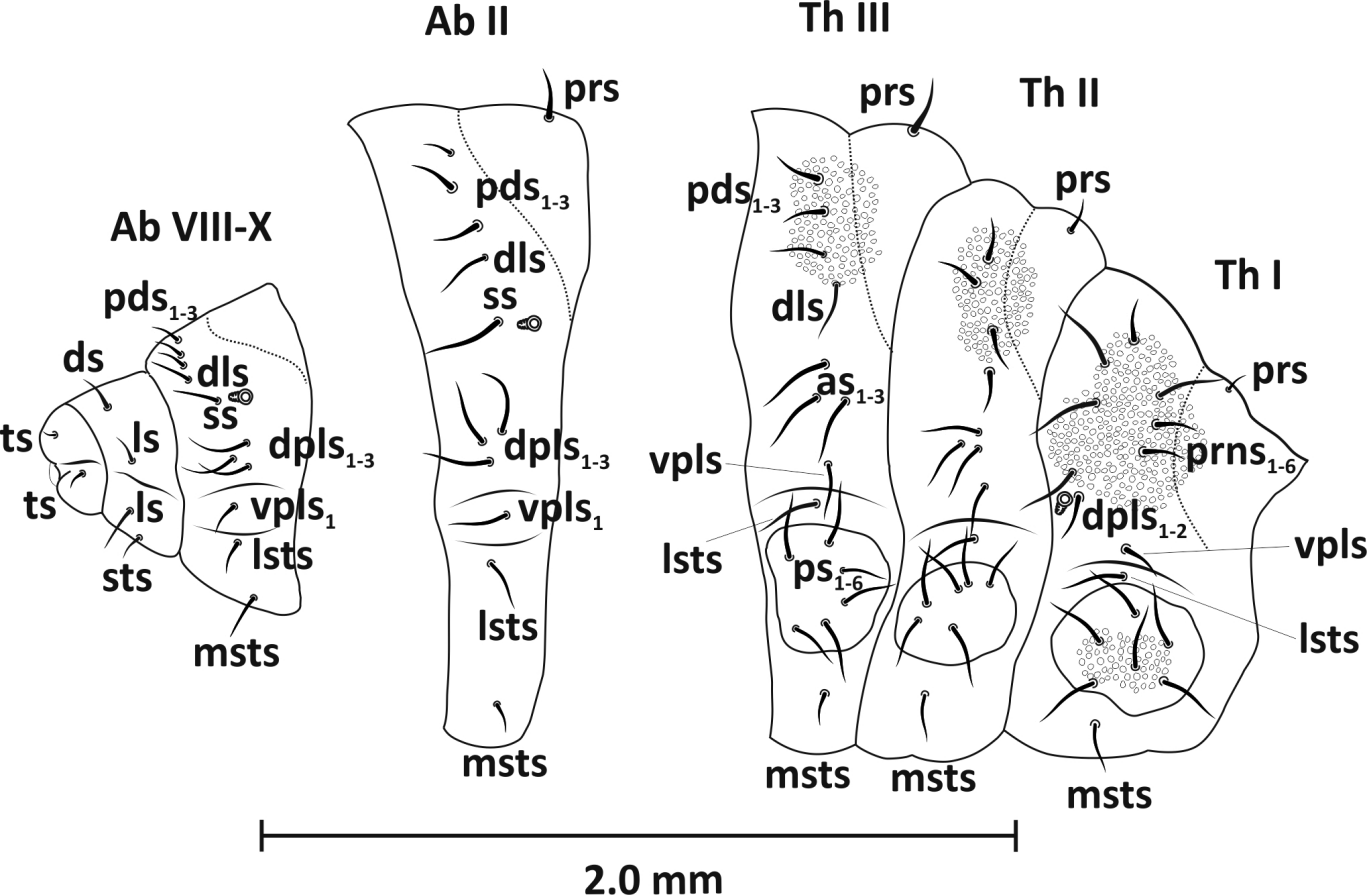
Mature larva (L_3_), selected segments, lateral view: *as* – alar seta, *dls* – dorsolateral seta, *dpls* – dorsopleural seta, *ds* – dorsal seta, *ls* – lateral seta, *lsts* – laterosternal seta, *msts* – mediosternal seta, *pds* – postdorsal seta, *prs* – prodorsal seta, *prns* – pronotal seta, *ps* – pleural seta, *ss* – spiracular seta, *sts* – sternal seta, *ts* – terminal seta, *vpls* – ventropleural seta.

*Thorax* (Figure 6). Pronotal shields sclerotised, meso- and metanotum each with two folds: pro- and postdorsum. Spiracle unicameral, situated at intersegmental septum near pedal lobe. Prothorax with twelve setae: pronotum with seven (long *prns1–6* and one short *prs*), epipleurum distinct with three setae of similar length (*dpls1–2* and one *vpls*). Sternum outside pedal lobe with two setae (one *lsts* and one *msts*). Pedal area on prothorax much more sclerotised than other two, with six setae of similar length (*ps1–6*) on all segments of thorax. Chaetotaxy of meso- and metathorax analogous, consisting of eleven setae: mesonotum with five setae (*pds1–3*, one *prs* and one *dls*). Epipleurum with four setae (*as1–3* and one *vpls*), sternum with two setae (one *lsts* and one *msts*). Pedal areas of meso- and metathorax unsclerotised.

*Abdomen* (Figure 6). Abdominal segments I-VII of similar shape. Tergites I-VII with two folds, prodorsum with one seta on ridge (*prs1*), postdorsum with five setae: four located dorsally (*pds1–3*, *dls1*) and one, longest seta (*ss*) on spiracular area. Epipleurum with four setae (*dpls1–3* and 1 *vpls*), sternum with two setae (one *lsts* and one *msts*). Segments I-VIII with unicameral spiracles, others (IX-X) without spiracles. Segment VIII with two folds, setae arranged as on segments I-VII, except for lack of seta *prs.* Segment IX with four setae, two on pleura (one *ds* and one *ls*) and two on sterna (one *ls* and one *sts*). Segment X with two small setae, one on pleura and one on sterna (both *ts*).

#### Description of pupa.

**Figure 7.**
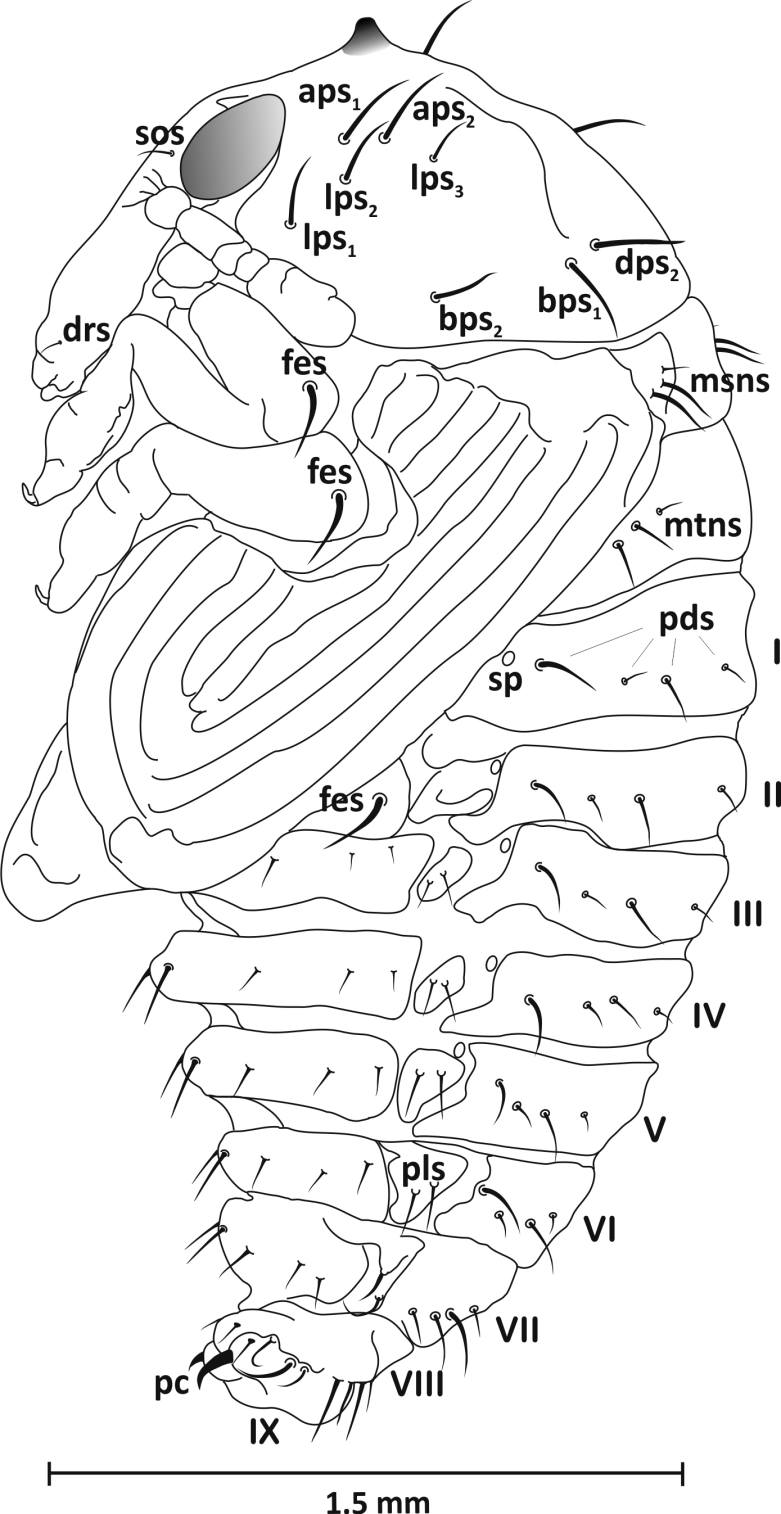
Pupa, lateral view: *aps* – apical pronotal seta, *bps* – basal pronotal seta, *dps* – discal pronotal seta, *drs* – distrirostral seta, *fes* – femoral seta, *lps* – lateral pronotal seta, *msns* – mesonotal seta, *mtns* – metanotal seta, *pds* – postdorsal seta, *pls* – pleural seta, *pc* – pseudocerci, *sos* – supraorbital seta, *sp* – spiracle.

**Figure 8.**
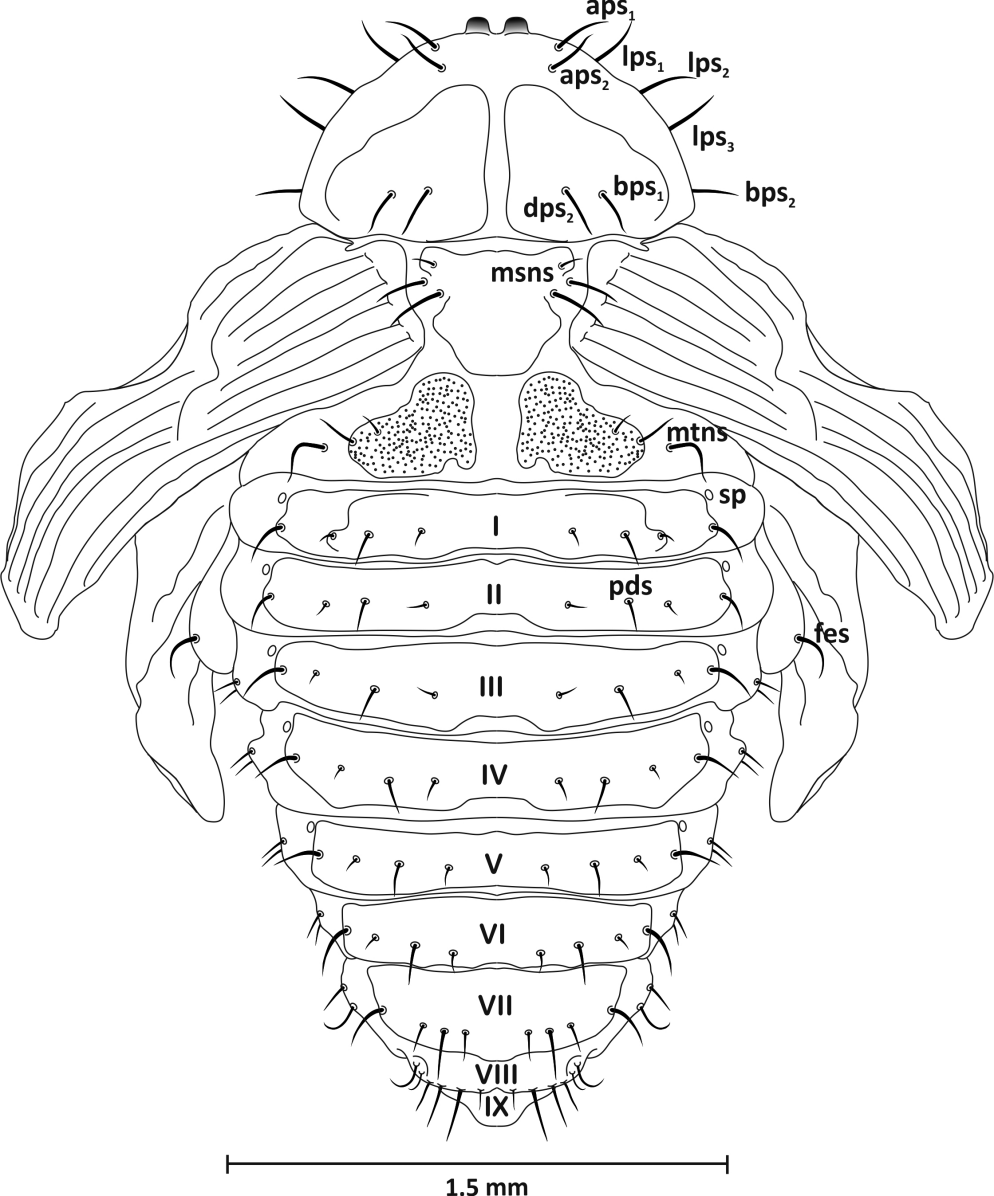
Pupa, dorsal view: *aps* – apical pronotal seta, *bps* – basal pronotal seta, *dps* – discal pronotal seta, *fes* – femoral seta, *lps* – lateral pronotal seta, *msns* – mesonotal seta, *mtns* – metanotal seta, *pds* – postdorsal seta, *sp* – spiracle,

**Figure 9.**
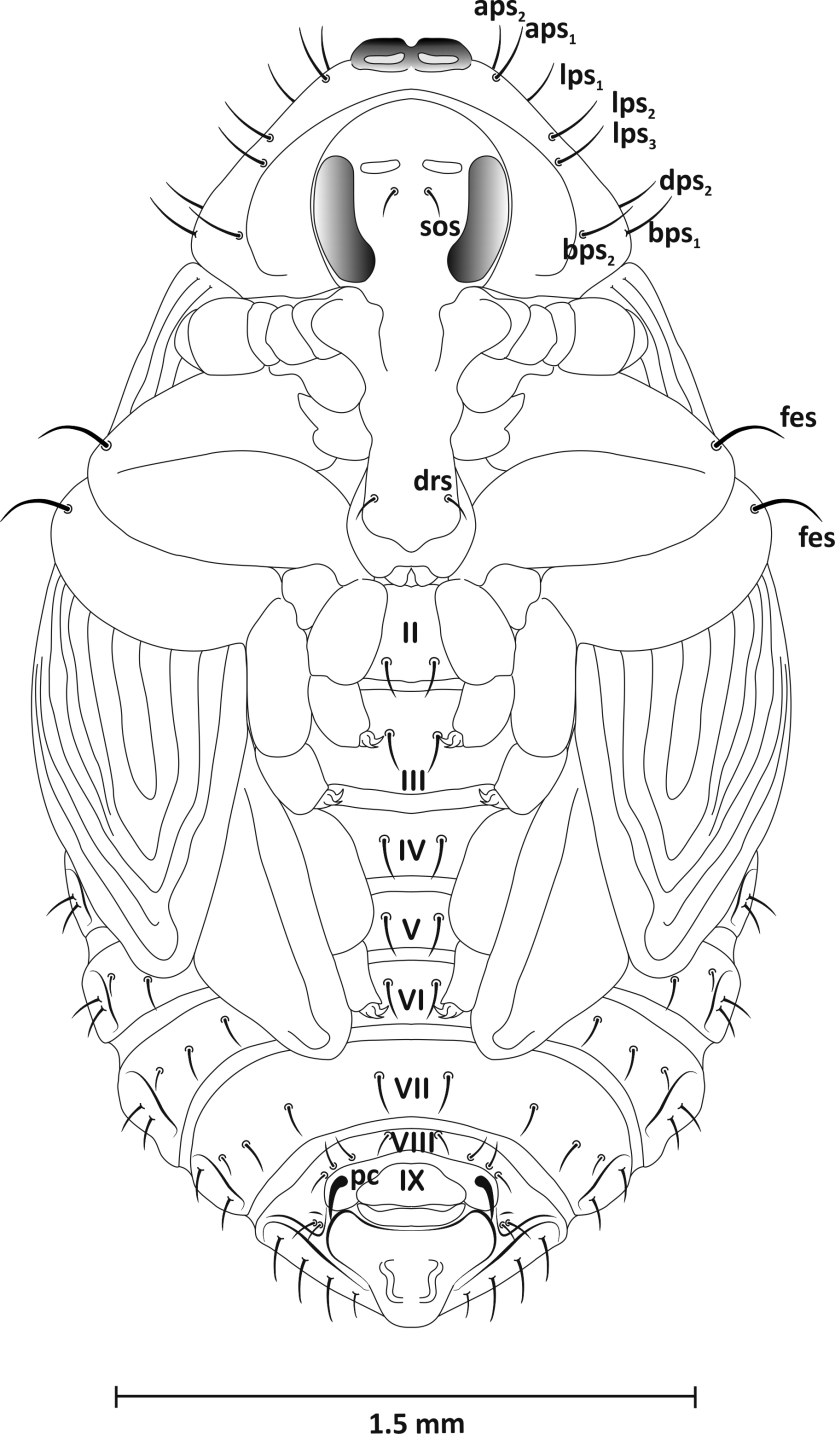
Pupa, ventral view: *aps* – apical pronotal seta, *bps* – basal pronotal seta, *dps* – discal pronotal seta, *drs* – distrirostral seta, *fes* – femoral seta, *lps* – lateral pronotal seta, *pc* – pseudocerci, *sos* – supraorbital seta.

(Figs 7–9, Table 2) *Measurements* (in mm). Body length: 3.32–3.40 (mean 3.35), width (between the apex of mesofemora) 1.90–1.98 (mean 1.92).

*Colouration.* Yellow-brown with distinct chaetotaxy.

*Head* (ventral view): rostrum reaches end of mesothorax, with one short seta (*drs*) apically. Head with distinct eyes and one seta (*sos*) at their inner edge. Antennae at base of rostrum. Massive. *Thorax*: pronotum wider than long, trapezoid-shaped, with two distinct, highly sclerotised, bare tubercles at anterior margin, with eight long setae: *aps1–2*, *lps1–3*, *dps2*, and *bps1–2* (Figs 7–9). Mesonotum longer than metanotum. Latter with two clearly visible scutellar shields posteriorly. Dorsal part of meso- and metanotum with three setae (*msns*, *mtns*) of unequal length located laterally. All femora with one long, thin seta (*fes*) located apically. *Abdomen*: tergites I–VII with four setae in one row slightly beyond midpoint of segment. Longest located near lateral outer margin, on segments I-V below spiracle entrance, on others (VI-VII) centrally. Of three remaining setae, two short – centrally located and third from middle of segment, second from centre somewhat shorter, but clearly shorter than outer seta. Segment VIII with four setae, shortest located medially, longest approx. three times longer than first. Segment IX with one distinct urogomphi, darker, bent back, highly chitinised (pseudocerci – *pc*) (Figs 7, 8). Sternites: segments I-VIII with four setae arranged in one row, of similar length except longest pair located medially (Figs 7, 9). Spiracles on abdominal segments I-V placed laterally, functional. Pleurites (III-VIII) with seta of equal length, slightly shorter and curved on segment VIII, pleurites (I-II) without setae (Figure 7).

**Table 2.** Differences of the pupa of *Rhinusaneta* and *R.bipustulata* based on the publications of Scherf 1964, Anderson 1973, Gosik 2010a, and on personal research.

	* Rhinusa neta *	* Rhinusa bipustulata *
Body	length 3.35 mm (mean), width (between the apex of mesofemora)1.92 mm (mean), yellow-brown	length 2.9–5.0 mm, width (between the apex of mesofemora) 1.50–2.60 mm, white or yellowish
Head	rostrum with 1 seta (*drs*), head with 1 seta (*sos*)	rostrum with 3 setae – 2 *drs*, 1 *es*, head with 2 setae (*brs*)
Thorax	pronotum: 8 setae: 2 *aps*, 3 *lps*, 2 *bps*, 1 *dps*, all femora with 1 long seta (*fes*)	pronotum: 9 setae: 2 *aps*, 3 *lps*, 2 *bps*, 2 *dps*, all femora with 2 long setae (*fes*)
Abdomen	dorsal part of segments I–VIII with 4 setae of unequal length in one row and 2 of setae located laterally on pleural area III–VIII	dorsal part of segments I–VIII with 4 setae of unequal length in one group (3) and 1 seta located laterally and 1 seta on pleural area I–VIII
	ventral part of segments I–VIII with 4 of setae, of which 1 medial is longer and 3 shorter, arranged in row	ventral part of segments I–VII with 5 short setae distributed in regular lines
	abdominal segment VIII with 4 setae of unequal length located dorsally, 1 lateral slightly shorter and 4 short setae located ventrally in regular line	abdominal segment VIII with 2 microsetae located dorsally and 2 short, thin setae located ventrally
	abdominal segment IX without setae	abdominal segment IX with 3 microsetae located ventrally
	pseudocerci (urogomphi) longer, clearly visible	pseudocerci (urogomphi) very short, poorly visible

#### Biological information.

After overwintering, adults emerge in May and June, depending on weather conditions in the year (Burakowski et al. 1997; Wilson et. al 2005). Initially, they feed on the vegetative parts of plants, and after the inflorescence has been formed they feed on the generative parts. After copulation, the females lays eggs into channels bored in the seed capsules and then uses excrement to seal the hole. One or two larvae were usually observed in one seed capsule. Approximately 10–12 days after eggs were laid eggs in the laboratory, L_1_ larvae appeared, which fed on the seeds without causing them to swell. The full larval stage lasted on average 29 days, followed by pupation. This stage lasted approximately 18 days. The full development cycle of the beetle in laboratory conditions averaged 58 days. Some larvae died in the seed capsules, having been attacked by parasitic hymenopterans of the superfamily Chalcidoidea, with parasitism reaching 20%. Species complete one generation per year and the new generation of adults emerge in August and September.

## Discussion

In the last ten years a number of studies by various authors have described the larval and pupal morphology of several taxa of Curculionoidea (Curculionidae, Apionidae): Lixinae (Gosik and Skuhrovec 2011; Gosik and Wanat 2014; Skuhrovec and Volovnik 2015; Trnka et al. 2015, 2016), Tychiini (Skuhrovec et al. 2014, 2015; Gosik et al 2017), Ceutorhynchinae (Gosik 2010b), *Bagous* (Gosik 2008), Hyperini (Skuhrovec 2006, 2007; Skuhrovec and Bogusch 2016), Entiminae (Gosik and Sprick 2013; Gosik et al. 2016), Molytinae (Sprick and Gosik 2014; Arzanov 2016) and Apionidae (Gosik et al. 2010; Wang et al. 2013; Łętowski et al. 2015). However, within the tribe Mecinini there are only two studies providing detailed descriptions of certain preimaginal stages of a few taxa (Gosik 2010a; Jiang and Zhang 2015).

In the genus *Rhinusa*, the most detailed morphology of immature stages in Europe can be found only for the species *R.bipustulata* in a study by Gosik (2010). For the remaining, fragmentarily described species, the only such studies are publications by Emden (1938) (larvae: *R.antirrhini*, *R.collina*, *R.neta*), Scherf (1964) (larvae: *R.antirrhini*, *R.bipustulata*, *R.collina*, *R.linariae*, *R.neta*, *R.tetra* and pupae: *R.antirrhini*, *R.bipustulata*, *R.collina*, *R.linariae*) and Anderson (1973) (larvae: *R.antirrhini, R.neta, R.tetra* and pupae: *R.antirrhini*, *R.neta*, *R.tetra*). For the very closely related genus *Gymnetron*, the immature stages of three species (*G.miyoshii* Miyoshi, 1922, *G.auliense* Reitter, 1907 and *G.vittipenne* Marseul, 1876) have recently been described (Jiang and Zhang 2015).

A study by Emden (1938) includes key characters by which the larvae of *R.neta* can be distinguished from *R.antirrhini* and *R.collina*. *R.neta* has a lighter, brownish-yellow head with a coarsely granulate frons. In addition, this species has 2–3 basal bristles of the maxillary mala that are more prominent than the others (Figure 5).

Scherf (1964) presents only few data on the biology of *R.neta* larvae, regarding its spectrum of host plants, feeding site, and the shape of the cecidia it causes. A work by Anderson (1973) presents graphics illustrating the frons of *R.neta* with an identical arrangement of setae (but unnumbered and unnamed) as in the present study, as well as characteristic, transverse asperities on its surface, making the L_3_ larvae easily distinguishable from *Rhinusaantirrhini*, which also feeds on the seeds of *Linaria*sp. (in which the frons of the L_3_ larva is smooth). Another distinctive character of *R.neta* is the presence of a long endocarina extending 4/5 of the length of the frons. On the epipleuron and pleuron of the abdominal segments (I–VIII) of the L_3_ larvae, there is a group of setae *dpls*1–3 and *vpls*1, which differs from the typical arrangement found in weevils (*dpls*1–2 and *vpls*1–2), but is confirmed in the key by Anderson (1973), according to which the three epipleural setae *dpls* distinguish *R.neta* from *R.antirrhini*, which has only two.

The number of setae on the dorsal surface of mandible within the Curculionidae family usually varies from 0 to 2. On this surface, there are also sensillae in the number of 0 to 3. The mandible of the discussed taxon generally resembles the system present in Ceutorhynchinae in terms of the setae and sensillae system (Scherf 1964), except that *R.neta* has three setae of similar length arranged in one line. The arrangement of the three setae at the mandible surface discussed in the paper is unique within this family.

In regard to the characters contained in the key by Marvaldi (2003) describing the length of a few setae on the frons (*fs4*, *des5*, and *les*), they are also well visible in this species, but setae *fs5*, *des3*, and *les2* are well developed as well. On the other hand, the lengths of setae *fs4* and *fs5*, which in Curculionidae sensu lato should be at least of similar length (or *fs4* should be longer), are not in agreement. In *R.neta* the pattern is reversed, as *fs5* is longer than *fs4*. A similar relationship between the lengths of these two setae is also reported in a study on *R.bipustulata* (Gosik 2010a), where *fs5* is designated as *fs3*.

As regards the biology of species, in addition to *Rhinusa* taxa, data on *R.pilosa* have been published as well (Gassmann et al. 2014). Phylogenetic research by Caldara et al. (2010), based on morphological data and host plants, indicates that the taxa most closely related to *R.neta* are *R.collina* (which is also confirmed in the key by Emden (1938)), *R.eversmanni*, *R.canescens* and *R.soluta*, but their preimaginal stages have not yet been described in detail. The similarities and differences between the larvae and pupae of *R.neta* and *R.bipustulata*, which has been studied in the greatest detail by Gosik (2010a), are presented below. The most significant differences between these species in the case of the L_3_ larval instars and pupae are shown in bold in Tables 1 and 2.

The information given in the two tables, grouping all available differences in the morphological structure of the L_3_ larva and pupa, can be used to prepare more detailed keys, both between the taxa given above and at the level of the tribe.

In the case of the pupae of the two taxa, the differences are in the number and location of the pairs of setae on the rostrum and head, number of pairs of setae on the prothorax, number of setae on the femora, location of the setae on the tergites, number of setae on the pleurites, number of setae on the sternites of segments I-IX, and length of the pseudocerci (or urogomphi).

Data presented by Wilson et al. (2005) indicate that in Canada the development cycle of *R.neta* lasts ca. 40–60 days, including 20–30 days for the larval stages and 10–15 days for the pupal stage.

## Supplementary Material

XML Treatment for Rhinusa neta
